# Diagnostic Value of Lingual Tonsillectomy in Unknown Primary Head and Neck Carcinoma Identification After a Negative Clinical Workup and Positron Emission Tomography-Computed Tomography

**DOI:** 10.3389/fonc.2018.00118

**Published:** 2018-04-20

**Authors:** Chad K. Sudoko, Marc A. Polacco, Benoit J. Gosselin, Joseph A. Paydarfar

**Affiliations:** ^1^Geisel School of Medicine at Dartmouth College, Hanover, NH, United States; ^2^Section of Otolaryngology, Audiology & Maxillofacial Surgery, Dartmouth-Hitchcock Medical Center, Lebanon, NH, United States; ^3^Norris Cotton Cancer Center, Lebanon, NH, United States

**Keywords:** squamous cell carcinoma, unknown primary, lingual tonsillectomy, transoral robotic surgery, transoral laser microsurgery

## Abstract

**Objective:**

Diagnostic rates of unknown primary head and neck carcinoma (UPHNC) using lingual tonsillectomy (LT) are highly variable. This study sought to determine the diagnostic value of LT in UPHNC identification using strict inclusion criteria and definitions to produce a more accurate estimate of diagnosis rate.

**Methods:**

In this retrospective chart review, records of patients who underwent LT for UPHNC were reviewed. Inclusion criteria included absence of suspicious findings on physical exam and positron emission tomography-computed tomography as well as negative biopsies after panendoscopy and palatine tonsillectomy. Following inclusion criteria, 16 patients were reviewed. A systematic literature review on LT for the workup of CUP was also performed.

**Results:**

LT was performed using transoral robotic surgery (TORS), transoral laser microsurgery (TLM), or transoral microsurgery with cautery (TMC). Following LT, primary tumor was identified in 4 patients out of 16. Detection rate by technique was 1/6, 2/7, and 1/3 for TORS, TLM, and TMC respectively. Postoperative bleeding occurred in three patients (19%); however, this was not related to the LT. Following literature review, 12 studies were identified; however, only 3 had enough data to compare against. All three studies had a cohort with suspicious findings on clinical exam. A total of 34 patients had a negative workup, with no suspicious findings on clinical exam and subsequently received an LT.

**Conclusion:**

This study suggests that LT should be considered initially in the diagnostic algorithm for UPHNC. This study can increase the patient size in this cohort by approximately 47%.

## Summary

- The rate of unknown primary detection from LT in the literature ranges from 18 to 90%, a range which may stem from small cohorts and heterogeneity of inclusion criteria.
- The rate of unknown primary detection from LT in this study was 25%.
- Bleeding was the most common complication and occurred in 19% of cases.
- LT should be advocated in the diagnostic algorithm for UPHNC to improve early detection rates.

## Introduction

Unknown primary head and neck carcinoma (UPHNC) presents as metastatic malignancy identified in a cervical lymph node without identification of primary origin on diagnostic examination (1, 2). When pathology is consistent with p16 positive squamous cell carcinoma (SCC), the oropharynx is the most likely source of origin (3, 4). Representing approximately 2–5% of all new head and neck malignancies, the primary site is eventually isolated to the palatine or lingual tonsils in 80–90% of patients (5, 6). When encountered, the first step in the work up of UPHNC is clinical evaluation involving a full history and physical exam, including flexible fiber optic laryngoscopy. Positron emission tomography-computed tomography (PET-CT) is often performed and carries a diagnostic rate of 7–38% (7, 8). Once imaging is complete, panendoscopy with tumor mapping is performed, with or without palatine tonsillectomy. Reported diagnostic rates of tumor mapping are approximately 20–50% when biopsies can be targeted with PET-CT; however, diagnostic rates markedly decrease to 9–29% when PET-CT is negative (5, 6, 9, 10).

The treatment of UPHNC entails neck dissection plus chemoradiation (CRT), neck dissection plus radiation, primary CRT, or primary radiation. Because a primary site cannot be identified, radiation fields are broad to target the entire oropharynx and hypopharynx, increasing risk of developing dysphagia, odynophagia, xerostomia, and dysphonia (1). Some studies have also suggested decreased survival in patients treated for UPHNC (11). When tumor location can be identified, radiotherapy can be targeted and intensity modulated to reduce side effects while still providing adequate treatment doses (12, 13).

The importance of reducing morbidity through primary site identification has spurred investigations into diagnostic protocol improvement. The addition of lingual tonsillectomy (LT) has become an increasingly prevalent adjunct due to improved diagnostic rates and low morbidity. In recent years, these procedures have been performed using transoral laser microsurgery (TLM) and transoral robotic surgery (TORS) as they enhance both visualization and mobility of tissue compared with traditional transoral instruments (14). With the addition of palatine and/or LT, primary site detection rates have been reported as high as 94% in addition to providing 100% 5-year disease free survival rates (15). With TORS, LT alone has reported diagnostic rates of 18–90% (1, 14, 16–19). This broad variation in diagnostic rates is likely due to the relative infrequency of UPHNC; studies assessing the diagnostic utility of LT contain small cohorts as well as differences in preoperative assessment, imaging, and surgical technique. We hypothesize that, with adherence to strict inclusion criteria for unknown primary based on negative clinical evaluation as well as PET-CT imaging without any suggestion of primary location, LT would result in a lower UPHNC detection rate than what is reported in most of the existing literature. In addition, we have performed a systematic review of literature to compare our results with similar studies.

## Materials and Methods

Approval for this study was obtained through the Dartmouth-Hitchcock Institutional Review Board. We reviewed the medical records of all patients presenting between February 2010 and May 2017 who had undergone LT and biopsy-proven metastatic SCC to cervical lymph nodes without an identified primary site. The patients were first evaluated in the outpatient setting and had negative findings on physical exam, flexible laryngoscopy, and PET-CT. To fit our inclusion criteria for unknown primary based on PET-CT, the imaging study had to be entirely negative without any suggestion of a primary site. Further inclusion criteria required patients to have undergone LT in addition to standard staging laryngoscopy/palatine tonsillectomy as part of their diagnostic workup. LT could have been performed concurrently with standard staging laryngoscopy/palatine tonsillectomy, or as a second procedure.

For all three approaches, the LT is performed by first making an incision along the lateral base of tongue and then carefully dissecting the lingual tonsillar tissue off the fold and tongue musculature. Dissection is carried down to the vallecula, the midline of the tongue base, and up to the circumvallate papillae and foramen cecum. The specimen is removed en bloc. The lingual tonsils are removed separately. After the tonsillar tissue is oriented with a suture, it is submitted for permanent pathologic analysis.

For TORS procedures, the da Vinci S or da Vinci Xi system is utilized. Exposure of the lingual tonsil is achieved by retracting the oral tongue forward and placing a Crowe–Davis retractor. A 30° 12 mm (da Vinci S) or 8 mm (da Vinci Xi) telescope, a combination of Maryland and Schertel graspers, and Bovie cautery attachments are utilized.

For TLM and transoral microsurgery with cautery (TMC) procedures, exposure is similar utilizing either the Lindholm operating laryngoscope or by retracting the tongue forward and placing a Crowe–Davis retractor. Visualization is achieved with an operating microscope (TLM and TMC) or laryngeal telescope (TMC). For TLM, a CO_2_ laser attached to a micromanipulator on the microscope is used whereas for TMC, a bovie cautery with an extended spatula tip is used.

### Search Strategy

A systematic review of published reports on LT for the workup of CUP was performed from June 2015 to March 2018 on MEDLINE, Cochrane Central Register, and CINAHL for all relevant English-language studies. Before June 2015, a systematic review from Fu et al. (19) was used. Keywords and subject headings specifying unknown primary, LT, SCC, and TORS or TLM were used to identify studies. Studies that included less than five patients were excluded. These studies also had to provide data on the number of patients who did not have suspicious findings on clinical workup and subsequently had an LT.

## Results and Analysis

Between February 2010 and May 2017, 16 patients met inclusion criteria and underwent TORS, TLM, or TMC LT. Of the 16 patients, 13 were male (81%). The ages ranged from 42 to 71, with a mean age of 59. Nine of the patients used tobacco and six of the patients reported daily alcohol use. One patient had a history of both basal cell carcinoma on his scalp and prostate cancer with no evidence of disease since prostatectomy in 2011. The remaining patients denied any previous malignancy. Demographic details of these patients are depicted in Table 1.

**Table 1:** Patient demographics and comorbidities.

	Age (years)	Tobacco abuse	Tobacco abuse	EtOH abuse	Comorbidities
Case 1	56–60	40 packs/year	40 packs/year	6 drinks/day	None
Case 2	56–60	None	None	None	None
Case 3	56–60	None	None	None	None
Case 4	61–65	40 packs/year	40 packs/year	None	T2DM, prostate CA, HTN, obesity
Case 5	66–70	40 packs/year	40 packs/year	None	Stroke, CAD, HTN, HLD, atrial flutter
Case 6	41–45	25 packs/year	25 packs/year	None	Anxiety, depression
Case 7	66–70	None	None	2 drinks/day	Hearing loss
Case 8	56–60	None	None	None	None
Case 9	51–55	10 packs/year	10 packs/year	None	None
Case 10	46–50	None	None	None	HLD, asthma
Case 11	51–55	20 packs/year	20 packs/year	1 drink/day	T1DM, osteoarthritis

### Clinical Workup

Fifteen patients presented with a level II and/or level III cervical node, and one patient presented with a level I lymph node. There were no localizing ENT symptoms, and all patients had negative findings on physical exam and flexible laryngoscopy. All FNA/core biopsies of the metastatic nodes identified SCC, 75% of which were p16 positive (Table 2). All patients underwent preoperative PET-CT, none of which showed evidence of primary tumor localization.

**Table 2:** Patient nodal status, surgery, and results.

	Nodal status	Surgery	Lingual tonsil	Concurrent neck dissection	Tumor size	Margins	p16
Case 1	R N2^a^	Transoral laser microsurgery (TLM) CO_2_	Positive	N	0.5 cm × 0.4 cm	Negative	Negative
Case 2	R N2	TLM CO_2_^b^	Negative	Y	N/A	N/A	Positive
Case 3	R N2	TLM CO_2_	Negative	N	N/A	N/A	Positive
Case 4	L N2	TLM CO_2_	Negative	Y	N/A	N/A	Positive
Case 5	L N3	TLM CO_2_^b^	Negative	N^c^	N/A	N/A	Negative
Case 6	L N2	TLM CO_2_	Negative	N	N/A	N/A	Positive
Case 7	L N1B	TLM CO_2_	Positive	N	Small foci	Negative	Positive
Case 8	R N2	Transoral robotic surgery (TORS)^b^	Negative	N	N/A	N/A	Positive
Case 9	R N2	TORS^b^	Negative	Y	N/A	N/A	Positive
Case 10	R N2	Transoral microsurgery with cautery (TMC)	Negative	N^c^	N/A	N/A	Positive
Case 11	R N2	TMC^b^	Positive	N^c^	Undetermined	Positive: deep margin	Positive
Case 12	R N2	TORS	Negative	Y	N/A	N/A	Positive
Case 13	L N2	TORS^b^	Positive	Y	0.3 cm^d^	Negative	Positive
Case 14	L N2	TORS	Negative	Y	N/A	N/A	Positive
Case 15	R N2	TORS^b^	Negative	Y	N/A	N/A	Negative
Case 16	R N2	TMC^b^	Negative	N	N/A	N/A	Negative

*^a^Liver and axillary metastasis*.

*^b^Bilateral lingual tonsillectomy*.

*^c^Neck dissection as a second procedure*.

*^d^In the largest diameter*.

### Surgical Approach

Lingual tonsillectomy was performed either during the standard staging laryngoscopy (7/16) or as a secondary procedure (9/16) with 8 patients receiving bilateral LT. Unilateral LT was performed in patients undergoing concurrent palatine tonsillectomy and was intended to minimize the small theoretical risk of oropharyngeal stenosis resulting from circumferential denuding of mucosa (20). Seven patients had their palatine tonsils removed for unrelated reasons before their current presentation. LT was performed using TORS (6/16), TLM (7/16), or TMC (3/16) with a detection rate of 25% (4/16). All detectable carcinomas were found on the ipsilateral side of the presenting lymph node. Of the four detectable carcinomas, one was 0.5 cm × 0.4 cm in size, a second was 0.3 cm in its largest diameter, a third was too small for measurement but had a small focus of cancerous cells, and the fourth extended into the deep margin with a surface epithelial to deep margin measurement of 0.5 cm. Three of the four carcinomas were p16 positive (Table 2).

### Complications

Although three patients develop bleeding postoperatively, none of the bleeding was associated with the LT. One patient required reoperation for bleeding from the palatine tonsil on postoperative day (POD) 6. This patient took apixaban on POD 5 due to a history of atrial flutter and stroke. A second patient had an expanding hematoma after neck dissection immediately postop and required operative management. A third patient presented to the Emergency Department for a palatine tonsillar bleed on POD 12, which was treated with topical silver nitrate cautery. All 16 patients were able to tolerate a soft diet at the first postoperative visit, and no patients had any significant weight loss. No patients required a G-tube following surgery. Three patients noted taste disturbances following surgery that improved on follow-up. There were no other complications from TORS, TLM, or TMC LT.

### Follow-Up

Follow-up ranged from 3.6 months to almost 7 years after LT with an average follow-up of 3.5 years. Following LT, seven patients received CRT, and four patients received only radiotherapy. Five patients elected to clinically monitor their status without chemotherapy or radiotherapy. Fourteen patients on last follow-up are alive without evidence of disease (Table 3). One patient developed recurrence in the right neck at levels IV and V, with spread to the liver and axilla 4 months after completion of radiotherapy. This patient was 1 of 4 cases that had a p16-negative carcinoma. She was a heavy smoker (40 pack/years) and heavy drinker (6 drinks/day) who continued to smoke after diagnosis and surgery. The final patient, in whom a primary cancer was never found, died from acute myeloid leukemia, 3 years after his initial LT with no evidence of recurrence of his UPHNC.

**Table 3:** Postoperative care and follow-up.

	Adjuvant therapy	Disease status	Surgery to last follow-up (months)
Case 1	RT	AWD	8.7
Case 2	RT	NED	23.6
Case 3	CRT	NED	21.7
Case 4	CRT	NED	12.3
Case 5	None	NED	7.1
Case 6	CRT	NED	52.6
Case 7	CRT	NED	38.2
Case 8	CRT	DSD	30.7
Case 9	CRT	NED	27.5
Case 10	None	NED	12.4
Case 11	RT	NED	5.5
Case 12	None	NED	84.3
Case 13	RT	NED	3.6
Case 14	None	NED	19.9
Case 15	None	NED	9.8
Case 16	CRT	NED	6.5

*RT, radiation therapy; CRT, chemoradiation; AWD, alive with disease; NED, no evidence of disease; DSD, died without disease*.

The systematic review from Fu et al. identified a total of eight studies, three of which had less than five patients, and two studies did not provide enough data on lingual tonsillectomies performed after a completely negative workup and were excluded. On the literature search from June 2015 to March 2018, four studies were identified. Of the four papers, one was the systematic review from Fu et al., and a second focused on radiotherapy characteristics and outcomes, and the last two did not include enough data. After review, three studies were included in our analysis.

## Discussion

Although not always possible, identification of the site of origin for unknown primary SCC is an essential goal for the head and neck surgeon. Radiotherapy increases patient morbidity with side effects such as xerostomia, dysphagia, and odynophagia; when treatments can be targeted to an identified location, patient morbidity is reduced. In addition, in select patients, treatment may consist of resection alone with avoidance of radiation depending on pathology and margin status. Thus, methods for increasing primary site identification are of great interest.

While there is currently no universal guideline for workup of unknown primary SCC of the head and neck, a national guideline is present in the United Kingdom. Typically, a workup starts with a full history and physical exam with flexible fiber optic laryngoscopy. PET-CT is often incorporated and presents a diagnostic rate ranging from 7 to 38% (7, 8). However, a major limitation of PET-CT is that tumors less than 1 cm in diameter are not reliably detected (21). In a study of 111 identified unknown primary tumors, the average diameter was 1.15 cm, and 57% of tumors were less than 1 cm in diameter (19). These data suggest that more than half of unknown primary tumors may be below PET-CT detection level, and their reported value may be an underestimation since tumors included were those able to be identified with imaging or panendoscopy. Despite this, Mackenzie et al. and the United Kingdom National Guidelines recommend that all patients presenting with confirmed cervical lymph node metastatic SCC and no identifiable primary should undergo PET/CT (22).

Once imaging is complete, panendoscopy with tumor mapping is traditionally performed. When PET-CT is able to provide targeted biopsies, diagnostic rates of tumor mapping range from 20 to 50% (5, 6, 9, 10). However, when physical exam and PET-CT are negative, diagnostic rates decrease to a range of 9–29% (5, 10). Depending on the institution, palatine tonsillectomy may be performed during panendoscopy and has been shown to provide cancer detection rates superior to biopsy of tonsillar tissue alone (23).

In recent years, LT performed with TLM and TORS has shown promising results at increasing UPHNC detection with rates ranging from 18 to 90%. The dramatic differences in detection rate are likely secondary to small cohorts in all studies, a consequence of infrequent presentation. We report an overall detection rate of 25% with LT. This detection rate is lower than most previously reported studies, but this is most likely secondary to our strict inclusion criteria. For the 16 patients in our study, there could be absolutely no suspicious findings on physical exam or PET-CT, and panendoscopic biopsies must all have been negative.

In a study by Mehta et al., LT with TORS yielded a 90% detection rate; however, 40% of these had positive BOT PET-CT findings, 20% of which were positive in the BOT ipsilateral to imaging, and 20% were positive on the contralateral BOT according to imaging (1). In a multi-institutional study by Patel et al., palatine and LT using TORS together resulted in 72.3% (34/47) tumors identified (14). For LT alone, the isolation rate was 42.6% (20/47). However, in this study 48.9% of patients had suspicious physical exam findings, 56.5% of which were confirmed to be cancer, and 34% of which had suspicious findings on PET-CT, 50.0% of which were confirmed to be malignancy. Nagel et al. performed LT on 14 patients, 57% (8/14) of which were positive, but it was not indicated whether imaging took place prior (17). It is likely that these rates of successful diagnosis are higher than our cohort secondary to inclusion of patients who had either suspicious exam findings or positive PET-CT findings, which would both increase the likelihood of included tumors being larger in size and thus easier to isolate.

However, a counterpoint would be that PET-CT carries a false positive rate up to 37%, thus patients with positive PET-CT findings could still be considered unknown primary (7). Furthermore, in a study by Durmus et al., of the 22 patients who underwent either a combination of palatine tonsillectomy with LT, LT alone, radical tonsillectomy, or base of tongue resection with TORS, lingual tonsils were positive in 4/22 (18%) of cases (18). This study presents a detection rate lower than that of our study, yet nine patients (40.9%) had PET-CT findings confirmed by surgical resection. Granted, most of these were positive palatine tonsils.

Most recently, a systematic review by Fu et al. reported LT identifying primary tumor in 72% (18/25) patients with no findings (19). The cohort size included in the systematic review was limited by heterogeneity of preoperative workups, definitions of unknown primary, and limited information regarding exact surgical techniques utilized in the literature. A prospective, multi-institutional trial utilizing homogenous preoperative workup, imaging, and surgical techniques would be required to present an accurate UPHNC diagnosis from LT.

In accordance with current changes in the epidemiological landscape in oropharyngeal SCC, the majority of neoplasms (75%) in this study were p16 positive (24). Although conclusions cannot be drawn based on low sample size, the detection rate of p16 positive tumors was higher than that of p16-negative tumors, rates being 75 and 25%, respectively.

Despite a large range of tumor isolation reported from LT, it is a useful adjunct in UPHNC identification and carries low risk of morbidity. The most common adverse event from LT is postoperative bleeding in 5% of cases (19). In this study, although three patients had bleeding events after surgery, none were related to the LT itself. Given that all reported LT UPHNC diagnostic rates exceed this value, it may be reasonable to perform LT at the time of panendoscopy with intent to save the patient a separate surgery and potentially expedite diagnosis and treatment.

The primary weakness of this study, one shared among all of the existing literature, is a small patient cohort. The uncommon presentation of unknown primary SCC in the head and neck in addition to our strict criteria were some of the reasons explaining the number of patients included in our study. After the literature review, only three studies, namely, Mehta et al. (1), Patel et al. (14), and Nagel et al. (17), provided enough information to determine an identification rate for LT after a negative clinical workup, including an absence of suspicious findings on PET-CT, panendoscopy with biopsies, and palatine tonsillectomy. When all suspicious findings were excluded, 43 cases from the three studies remain, with 34 receiving LT (Table 4). Although a small patient cohort is present, it is similar to other studies and increases the number of patients with a negative clinical workup and subsequent LT by almost 50%. Another potential weakness of this study is that three differing resection techniques were utilized, although successful identification of a primary was achieved with each technique. Of note, seven patients had a neck dissection concurrent with the tonsillectomy, three patients had a neck dissection as a second procedure, and six elected to not have a neck dissection. The differences in practice patterns were largely a result of recommendations from tumor board, dependent on the characteristics of the metastatic node and if the patient elected to receive adjuvant therapy. Finally, in eight patients, LT was only performed on the side of the presenting nodal metastasis. While unilateral LTs were performed to reduce patient morbidity, reported rates of unknown primary in the contralateral lingual tonsil are 10% (18).

**Table 4:** Proportion of patients without suspicious findings on diagnostic workup and identification in the lingual tonsil.

Reference	Institution	Proportion with suspicious findings	Proportion without suspicious findings on PET/CT, EUA with biopsy, and palatine tonsillectomy	Identification in lingual tonsil after negative workup
Mehta et al. (1)	University of Pittsburgh Medical Center	4/10	6/10	5/6

Patel et al. (14)	University of Washington Medical Center	32/47	15/47	8/14
	MD Anderson Cancer Center			
	University of Alabama Birmingham			
	University of Texas Medical School at Houston			
	Johns Hopkins Hospital			
	Oregon Health & Sciences University			

Nagel et al. (17)	Mayo Clinic Arizona	30/52	22/52	8/14

Sudoko et al. (the current study)	Dartmouth-Hitchcock Medical Center	0/16	16/16	4/16

The rate of unknown primary detection from LT in patients with a negative PET/CT was 25%. The rate of unknown primary detection from LT in the literature ranges from 18 to 90%, although cohort size is a limitation of all existing studies. Nevertheless, LT improves unknown primary site identification and carries low risk of complications and therefore should be advocated in the diagnostic algorithm for UPHNC to minimize treatment morbidity.

## Ethics Statement

This study was carried out in accordance with the recommendations of the Dartmouth-Hitchcock Institutional Review Board. Due to the retrospective nature of the study, the Institutional Review Board waived the necessity to obtain patient consent as long as all patient identifiers are kept confidential.

## Author’s Note

This manuscript was presented at the Fifth Annual Surgical Trainees Advancing Research Symposium, Dartmouth-Hitchcock Medical Center, Lebanon, NH, USA, April 2017.

## Author Contributions

CS, MP, BG, and JP: helped with study design; data acquisition, analysis, and interpretation; and coauthored manuscript.

## Conflict of Interest Statement

The authors declare that the research was conducted in the absence of any commercial or financial relationships that could be construed as a potential conflict of interest.
